# Roots to the rescue: how plants harness hydraulic redistribution to survive drought across contrasting soil textures

**DOI:** 10.1007/s44307-024-00050-8

**Published:** 2024-11-25

**Authors:** Shenglan Sha, Gaochao Cai, Shurong Liu, Mutez Ali Ahmed

**Affiliations:** 1 School of Agriculture and Biotechnology, Shenzhen Campus of Sun Yat-sen University, 518107 Shenzhen, China; 2 Root-Soil Interaction, TUM School of Life Sciences, Technical University of Munich, 85354 Freising, Germany

**Keywords:** Hydraulic redistribution, Drought, Soil texture, Root hydraulic conductivity, Water potential gradient

## Abstract

Hydraulic redistribution (HR) is a critical ecological process whereby plant roots transfer water from wetter to drier soil layers, significantly impacting soil moisture dynamics and plant water and nutrient uptake. Yet a comprehensive understanding of the mechanism triggering HR and its influencing factors remains elusive. Here, we conducted a systematic meta-analysis to discuss the influence of soil conditions and plant species characteristics on HR occurrence. The threshold of HR ranges from -1.80 to -0.05 MPa, with soil hydraulic conductivity between 1.51 × 10^–13^ and 6.53 × 10^–5^ cm s^−1^ when HR occurs. HR is influenced by various factors. Soil texture plays a pivotal role, with loamy soils promoting HR more effectively than sandy and clay soils. Plant root structure and hydraulic conductivity significantly influence HR occurrence, where HR is more prevalent in deep-rooted tree species with larger root canal diameters and dimorphic roots. Additionally, mycorrhizal fungi enhance HR by expanding root uptake area, reducing water transport distances and improving soil structure. However, adverse soil conditions, inadequate plant physiological regulatory capacity, or methodological limitations can hinder HR detection. The findings highlight that HR is more likely to occur where there is a significant water potential gradient, appropriate root-soil contact, and low nocturnal transpiration. Plants can effectively replenish the water in dry root systems under drought conditions by HR by increasing the water potential of root systems to maintain normal physiological functions. Our study identifies key factors influencing HR, offering a comprehensive framework for future research aimed at improving plant drought resistance and refining ecohydrological models.

## Introduction

**Fig. 1:**
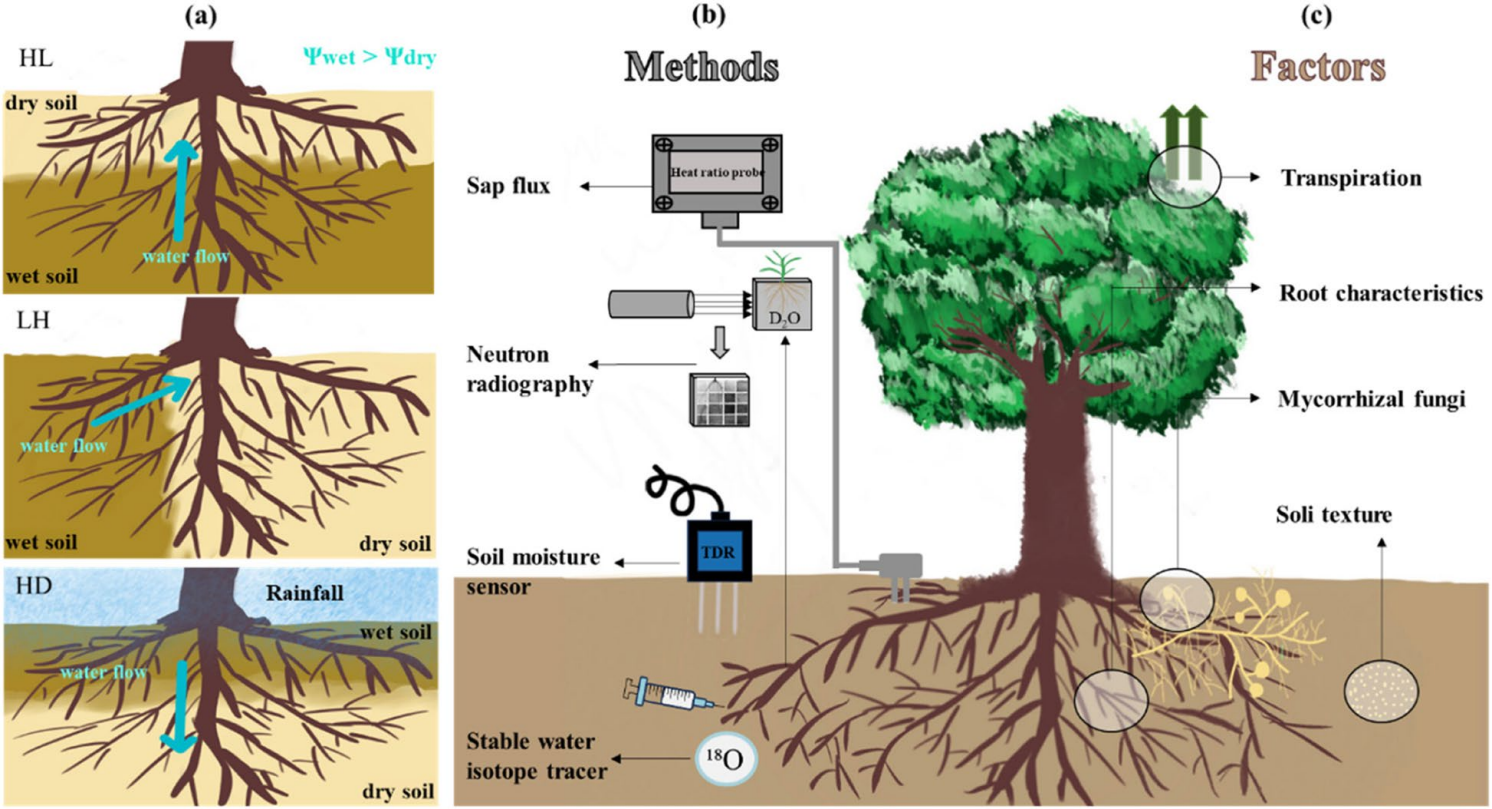
Types, detection methods, and influencing factors of hydraulic redistribution (HR). **a** Three common HR phenomena. Hydraulic lift (HL) occurs when the upper soil is relatively dry and the lower soil is relatively wet. Lateral redistribution (LR) occurs in soil layers at the same depth with a water potential gradient. Hydraulic descent (HD) often occurs after rainfall when the upper soil is wetter than the deeper soil. Some factors affecting the occurrence of HR in plants and methods of detecting HR occurrence (right). **b** A variety of technical methods for the detection of HR. The specific neutron radiography technique used in the detection method is described in (Ahmed et al. 2016). **c** Some factors affecting the occurrence of HR in plants

In agricultural production, water is a vital guarantee for crop yield and quality by participating in various physiological processes such as plant nutrient absorption, photosynthesis, and transpiration (Fang and Xiong 2015; Sharma and Kumar 2023). However, industrialization and population growth are accelerating global warming, which has intensified droughts. These droughts are damaging crop production and pose a major threat to global food security (Huang et al. 2014, 2015; Xu et al. 2021). Facing extreme drought conditions, optimal soil moisture is of critical importance as it directly impacts plant water absorption and utilization efficiency, thereby bolstering crop drought tolerance (Liste and White 2008; Alagele et al. 2021; Sharma and Kumar 2023; Vadez et al. 2024). Plants can adapt physiological mechanisms to cope with drought conditions, by actively closing stomata to reduce water transpiration, adjusting cell fluid osmolality to absorb more water from the outside, and by redistributing water from wet soil to dry soil through their roots, a process called hydraulic redistribution (HR) (Hultine et al. 2004; Tardieu et al. 2018; Sharma and Kumar 2023). When subjected to drought stress, plants respond by reallocating water from wetter sites to the stressed root zones (Hafner et al. 2020; Werner et al. 2022). The primary driving force for this water movement is the soil matric potential, however, osmotic potential within the soil also contributes to this movement (Prieto et al. 2012). The former determines the adsorption and retention of water in the soil matrix, whereas the latter one influences both the velocity and path of water flow through the root system. Water flows from regions of higher water potential to regions of lower water potential until equilibrium is achieved and potential differences are balanced. This phenomenon was first observed in greenhouse experiments (Breazeale 1930; Breazeale and Crider 1934). However, it was not until half a century later that diel soil water potential fluctuations were discovered during nocturnal experiments on the shrub *Artemisia tridentata* and formally proposed as "hydraulic lift" (HL) (Richards and Caldwell 1987). Furthermore, when discrepancies in soil water potential occur across different horizontal locations, water can be transported from areas of higher water potential to those of lower potential through lateral roots (Pitono et al. 2020). This process, known as lateral redistribution (LR), depends on the spatial heterogeneity of soil water potential (Smart et al. 2005; Bleby et al. 2010). A third hydraulic redistribution (HR) phenomenon (after HL and LR) is the downward transport from shallow wet soil to deep dry soil after rainfall, that is, hydraulic descent (HD) (Fig. 1a) (Burgess et al. 1998, 2001).

HR is a passive process driven by the water potential gradient between soil layers. It typically occurs at night when transpiration is very low, although it can also occur during the day if the water potential gradient driving force is sufficiently large (Brooks et al. 2006; Bleby et al. 2010). HR may – in principle – occur between soil layers in which no roots are present. In most cases, however, plant roots drive HR by enhancing water potential gradients and by providing the main and fastest water transport paths through soil. Plant roots need to be connected across the vertical or horizontal gradients to function as water transport pipes (Neumann and Cardon 2012; Prieto et al. 2012; Bazihizina et al. 2017; Hafner et al. 2017; Lee et al. 2018). HR has been observed across different climates and ecosystems, for example in sandhill community (Espeleta et al. 2004), semiarid deserts (Ryel et al. 2002; Hultine et al. 2003, 2004; Hill et al. 2021), tropical forests (Meinzer et al. 2004; Oliveira et al. 2005), tropical (Scholz et al. 2008) and semi-arid savanna (Scott et al. 2008; Lee et al. 2018), semi-arid Mediterranean climate (Prieto et al. 2010) and subtropical monsoon climate (Wei et al. 2022). The species involved range from herbaceous (Leffler et al. 2005; Meunier et al. 2018; Hayat et al. 2020; Werner et al. 2022) to woody shrubs and trees (Hao et al. 2009; Prieto et al. 2010; Bogie et al. 2018; Wang et al. 2022), occurring in different soil types (Yoder and Nowak 1999; Liu et al. 2023). Since HR is a complex process within the soil, its assessment and measurement are inherently challenging.

To accurately observe and predict the mechanism of HR, a variety of techniques have been employed (Fig. 1b). One technique involves placing soil moisture sensors (Time Domain Reflectometry, TDR; Frequency Domain Reflectometry, FDR) at the root distribution points, allowing for the real-time monitoring of soil water content variations on diurnal and nocturnal timescales (Leffler et al. 2005; Howard et al. 2009; Pitono et al. 2020; Van Dusschoten et al. 2020). Another approach uses sap flux technology (Heat Ratio Probes; Heat Pulse Velocity, HPV) to measure root sap flow, thereby estimating the amount and direction of water movement in roots (Hultine et al. 2003; Alagele et al. 2021; Liu et al. 2023). A further technique employs stable water isotope tracers, such as the hydrogen isotope (^2^H) and the oxygen isotope (^18^O), which are injected into local specific areas of the root system to track water flow through soil and plants (Hafner et al. 2017; Bogie et al. 2018; Meunier et al. 2018; Fan et al. 2023). A recent approach combines neutron radiography with heavy water D_2_O to visualize water and root distributions in soil (Hayat et al. 2020).

The occurrence of HR has attracted increasing attention due to its influence on plant, community, and ecohydrological aspects (Neumann and Cardon 2012; Prieto et al. 2012; Barron‐Gafford et al. 2017, 2021). For plants, HR affects water balance in dry soil environments, which helps them withstand long-term drought and prolongs the life of fine roots (Prieto et al. 2012). Redistributing water into shallow soils can reduce soil drying rates, and promote microbial activity that could potentially improve soil nutrient levels, while promoting plant transpiration and nutrient uptake (Neumann and Cardon 2012; Sardans and Peñuelas 2014). Sardans & Peñuelas (2014) have indicated that HL can increase the water content of shallow soils by 28% to 102% (Brooks et al. 2006; Hao et al. 2010; Brooksbank et al. 2011; Warren et al. 2011). For instance, Kurz-Besson et al*.* (2006) used stable isotopes to estimate the contribution of HL water from *Quercus suber L.* trees in a Mediterranean climate, finding that 17–81% of the next day's transpiration was from HL water. Liu et al. (2021) employed the sap flow method to assess the root system of *Populus tomentosa* and identified evaporation-driven hydraulic redistribution (EDHR). Under extreme drought conditions, EDHR effectively replenished water to dry roots and significantly increased root water potential by 38.9% to 41.6%, thereby highlighting the exceptional drought ameliorating effect of EDHR in plants (Liu et al. 2023). At the community level, water released into the soil by deep-rooted plants exhibiting HR can be utilized by neighboring species (Prieto et al. 2012; Gerjets et al. 2021). Waren et al*.* (2008) found that ectomycorrhizae associated with root seedlings can acquire a substantial quantity of HR water from the roots of large trees and may transfer water through a common mycorrhizal network, connecting trees and seedlings. The sharing of water between different species through HR has important implications for the structure and function of plant communities. Furthermore, a North Carolina survey revealed that ecosystem transpiration increased by 30–50% due to HR mediation, significantly contributing to both total and net primary productivity by maintaining the carbon balance of the entire ecosystem (Domec et al. 2010).

The hydrological cycle that plants participate in through transpiration plays a crucial role in maintaining the carbon balance of ecosystems (Eamus 2003). Any factor that enhances water absorption and utilization by plant roots contributes to the carbon storage capacity and overall productivity of forest ecosystems (Domec et al. 2010). Therefore, an in-depth analysis of HR mechanisms closely related to plant transpiration and drought stress response in terrestrial ecosystems and agroecosystems is of great importance (Liste and White 2008). Given that HR only occurs under specific environmental conditions and is influenced by multiple factors such as species, soil, and climate (Fig. 1c) (Neumann and Cardon 2012; Hafner et al. 2017; Yang et al. 2022), we conducted a meta-analysis to explore these dynamics. Our analysis identified key factors impacting HR, including soil texture and depth, plant species, and water potential threshold intervals when HR occurs. We examined the effects of soil texture, root structure and distribution characteristics, plant transpiration, and mycorrhizal fungi on HR, and also addressed the challenges in detecting HR. Additionally, we investigated the potential variations of plant species to trigger HR, thereby providing a comprehensive understanding of plant responses to environmental stress.

## Threshold for water potential that triggers HR

The spatial variability of various environmental and meteorological factors leads to the heterogeneous distribution of soil water, and plants typically utilize HR to cope with this variability (Thomas et al. 2020). The water potential gradient between the drier and wetter soil layers is a key driver of HR, influenced by a number of factors, including soil water heterogeneity, rainfall and irrigation, transpiration, and root water uptake (Scholz et al. 2002).

Studies have reported that HR tends to occur when the soil water potential gradient between these layers is approximately −0.8 to −1.2 MPa (Ishikawa and Bledsoe 2000; Scholz et al. 2008; Zapater et al. 2011; Ke et al. 2022). Although a larger water potential gradient is generally more favorable to the HR occurrence, a large amount of water was shown to redistribute even with a small water potential gradient (Prieto et al. 2010; Hafner et al. 2020). It’s worth noting that not all water potential gradients driving HR are due to differences in water content (Yang et al. 2019). For instance, a study in Florida comparing coastal tall trees and inland dwarf trees showed that HR in dwarf trees, as detected by reverse sap flow, was driven by differences in salinity in saturated soils, rather than water content alone (Hao et al. 2009). However, the amount of instances of HR caused by osmotic gradients resulting from the difference in soil salinity is very limited and further research is needed in this area (Bazihizina et al. 2017).

**Fig. 2:**
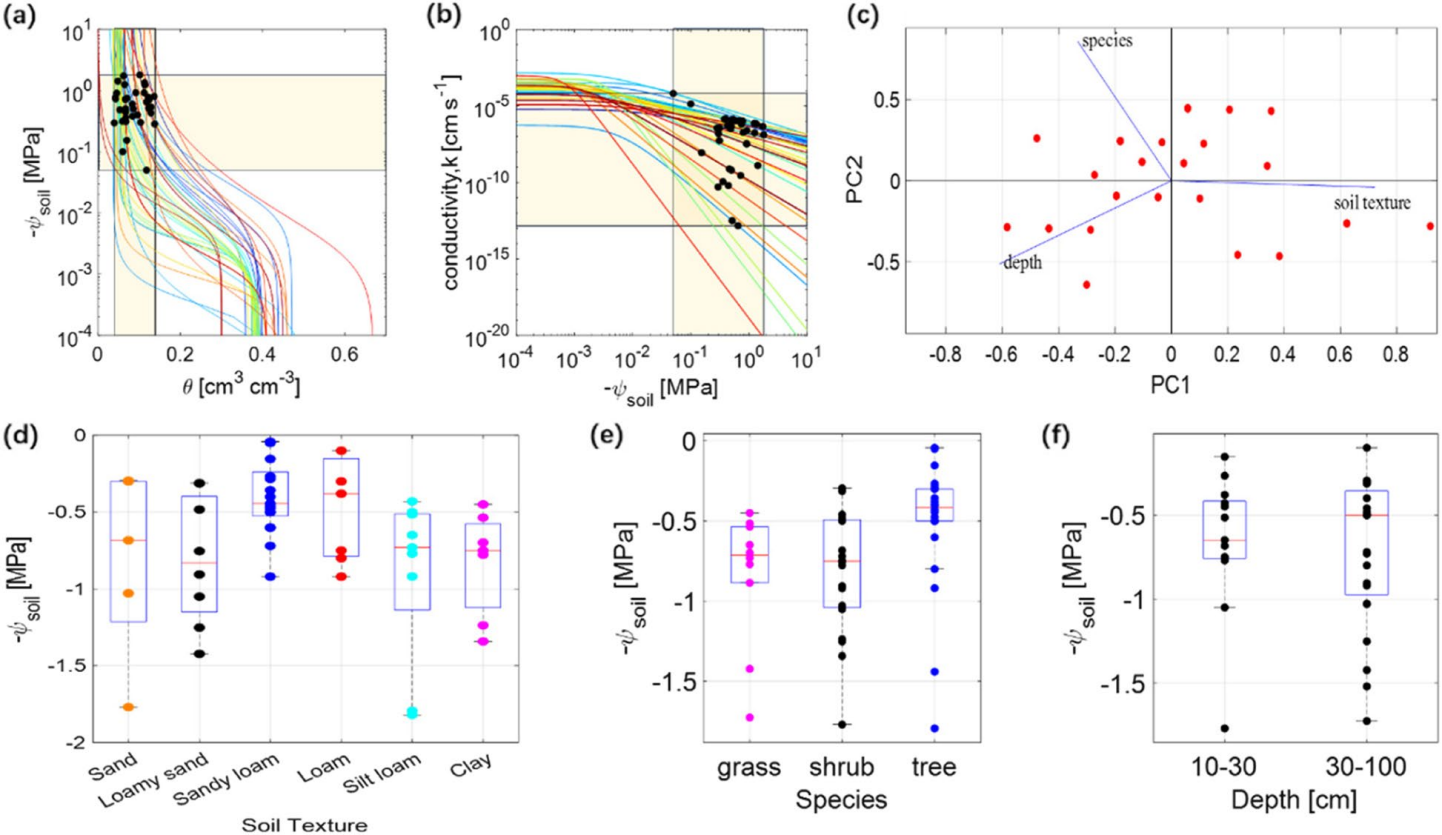
Values of soil water potential when HR occurred across different soil textures, plant species, and soil depths. **a** The soil water retention curves and the distribution of soil water potential at which HR occurred. **b** The soil hydraulic conductivity curve of contrasting soil textures. In (**a**) and (**b**), the yellow area represents the interval where the hydraulic redistribution variable occurs and the black solid circle represents the observed value at the onset of hydraulic redistribution. Some studies only provided curve parameters without illustrating the water potential value of HR, so there were no data points on these curves. **c** Principal component analysis of soil texture, plant species, and soil depth. Principal Component 1 (PC1) represents the first principal component, which is the main source of variation in the data. Principal Component 2 (PC2) represents the second principal component, which represents the second most important source of variation in the dataset, second only to PC1 in explaining total variation. **d-f** Effects of plant species, soil texture, and depth on HR, where the solid circle represents a single observation

Considering the complexity of factors influencing HR, we conducted a meta-analysis to determine the water potential threshold in the drier soil layers at which HR occurs under different soil textures, depths, and plant species since the water potential in wetter layers is much higher (less negative) (Fig. 2a). The analysis showed that the water potential in the drier soil layer (mostly in the shallow layers) where HR occurs is typically within the range of −0.05 and −1.80 MPa (Ishikawa and Bledsoe 2000; Meinzer et al. 2004; Brooks et al. 2006; Querejeta et al. 2007; Domec et al. 2010; Neumann and Cardon 2012). For example, in the upper 0–20 cm soil of Douglas fir forests in the southern region of Washington, the occurrence of HR is initiated when the soil water potential dried to −0.4 to −0.7 MPa (Dawson 1993; Brooks et al. 2006). However, HR can also occur at higher soil water potentials (Fig. 2a) (Domec et al. 2004; Warren et al. 2005). In the Pacific Northwest forest ecosystem, the water potential threshold for HR was approximately −0.05 MPa in the old-growth forest (Domec et al. 2004). Similarly, HL can be established when the water potential of the upper 15–60 cm of the coniferous forest roots in the Pacific Northwest dropped to −0.05 MPa (Warren et al. 2005). An investigation in the southeastern USA on four tree and two herbaceous species in waterfall line dunes revealed that HL was exclusively evident when soil water potential reached −0.2 MPa or below (Espeleta et al. 2004). These results highlight the variability in HR initiation thresholds depending on species, soil texture and environmental factors. However, HR consistently occurs within a defined range of soil water potentials in the drier layers, providing valuable insights into the conditions that most favor water redistribution. Understanding these thresholds can help predict when and where HR is likely to occur, providing a framework for improving water management strategies in ecosystems vulnerable to drought.

## Factors impacting hydraulic redistribution occurrence

### Soil texture

Soil texture affects HR by influencing soil hydraulic conductivity, which in return impacts the ability of the soil to transport water (Neumann and Cardon 2012; Liu et al. 2023). It also determines water-holding capacity and permeability, thereby affecting the transport, absorption, and availability of water to roots (Scharwies and Dinneny 2019). Recent studies indicated that soil texture influences the quantity of water retained in the soil and the amount released by plant roots (Liu et al. 2023). Our principal component analysis (PCA) of variables included things such as plant species, soil texture, and depth, and identified soil texture as the most influential factor on HR (Fig. 2c). According to our statistics on the relevant literature, when HR occurs, the soil hydraulic conductivity ranges from 1.51 × 10^–13^ to 6.53 × 10^–5^ cm s^−1^ (Fig. 2b). In some studies, higher hydraulic conductivity of coarser soils was shown to accelerate the flow of water from the surface layer to the deep layer, increasing the water potential of the deep soil and thus creating a more conducive environment for HL (Yu and D’Odorico 2014). For instance, Zou et al. (2005) found that HL in savanna tree-shrubs clusters occurred more frequently in groves with deep sandy soils than in clusters with shallow clay soils. However, in soils with particularly high hydraulic conductivity, the soil water potential gradient cannot be maintained for long, resulting in relatively lower HR occurrence. For example, in the sandy soils on the Swan Coastal Plain to the north of Perth, the high conductivity and permeability of water caused downward HR to be unsustainable because the required water potential gradients between soil depths could not be maintained (Burgess 2000). A similar phenomenon was observed in the well-drained soils of the Cerrado in central Brazil (Scholz et al. 2008). Thus, some authors proposed that the promotion of HR occurrence in sandy soils is less than that in other soil textures (see also the section: Challenges in detecting hydraulic redistribution) (Neumann and Cardon 2012). Coarser soils have higher soil resistance than finer soils, which limits the exchange of water between plant roots and soils (Liu et al. 2023). A negative correlation was observed between the frequency of HL and the percentage of sand at 0.35 m depth in the soil across native plants in the Mojave Desert, indicating that the coarser soil texture is not conducive to the occurrence of HL to some extent (Yoder and Nowak 1999). In controlled experiments on the HL of cotton, the root length density in the sandy soil subsoil was relatively low compared to the clay topsoil, so less water may be lifted to the roots in the topsoil (Wang et al. 2009).

In contrast, finer soils like loamy soils, with intermediate hydraulic conductivity and permeability, are well-suited to facilitate the formation of water potential gradients between shallow and deep (or dry and wet) soil layers after rainfall, thereby promoting the HR (Ryel et al. 2004; Oliveira et al. 2005). Specifically, the magnitude of HR in loam was found to be significantly higher than in sand or clay in Fig. 2d (Yang et al. 2022). Loam soils strike a balance between water retention and drainage, allowing them to sustain the gradients required for HR longer than sandy or clay soils. For instance, Prieto et al. (2010) found that five shrubs in Spain redistributed 3.6 times more water in loam than in sandy soils, suggesting that loam soils are more favorable for shrub water absorption. This suggests that loam soils are more conducive to the occurrence of HR than sandy soils and clay soils to some extent (Schymanski et al. 2008). However, it is important to note that this enhanced HR in loam is contingent upon specific soil moisture conditions that allow for the necessary water potential gradients to develop. While coarse soils may enable HR under specific conditions, their overall tendency to rapidly dissipate water potential gradients and limit root-soil water exchange generally makes them less favorable for sustained HR compared to finer-textured soils like loam. Therefore, the overall effectiveness of HR in loam soils is a result of their ability to balance water retention and permeability, making them particularly conducive to HR in appropriate moisture regimes. These findings underscore the critical role of soil texture and soil moisture conditions in influencing HR dynamics, with broader implications for plant water use efficiency.

### Root characteristics

Root characteristics have been widely recognized as key internal drivers of HR (Yu and D’Odorico 2014). These included various root structural, morphological, and physiological traits (Yang et al. 2022), with root hydraulic conductivity and root distribution playing crucial roles in facilitating the movement of water within the soil and plant system (Cai et al. 2022), thereby influencing both the efficiency and occurrence of HR (Cai et al. 2018b).

#### Root hydraulic conductivity

The variation in plant root hydraulic conductivity has a significant influence on the occurrence of HR (Neumann and Cardon 2012; Bazihizina et al. 2017; Liu et al. 2023). The contribution of water transport within the root system was found to be significantly greater than that of water movement between soil layers (Richards and Caldwell 1987; Ryel et al. 2002; Brooks et al. 2006). Ryel et al. (2002) used model simulations to show daily water movement through transpiration, unsaturated soil and root hydraulic lift in each soil layer over a period of 100 days, and found that water movement through root hydraulic lift was much greater than water movement through unsaturated soil in most soil layers. Some studies indicated that a decrease in root hydraulic conductivity leads to a reduction in HR (Domec et al. 2004; Warren et al. 2007; Prieto et al. 2010). For instance, in the lateral roots of trees within the Neosavanna ecosystem, a decrease of about 45% in hydraulic conductance loss was accompanied by an increase of over 350 kg m^−2^ day^−1^ in reverse sap flow (Scholz et al. 2008). Similarly, Warren et al. (2007) found that, under relatively dry soil conditions, a substantial soil water potential gradient was sufficient to drive HR, after reaching a peak, reduced hydraulic conductance due to root embolism resulted in a decline and finally suspension of HR. Additionally, the authors also discovered that seasonal changes in root hydraulic conductivity had a considerable influence on HR (Warren et al. 2007). During the wet season, vigorous root growth and sufficient soil moisture enhanced the root hydraulic conductivity, thus promoting HR. Conversely, during the dry season, reduced root activity and lower soil moisture led to a reduction in hydraulic conductivity thus limiting the efficiency of HR (Warren et al. 2007).

Root hydraulic architecture and its components also impact HR. Both axial and radial hydraulic conductivities affect the flow rate of water redistribution (Bazihizina et al. 2017). Axial conductivity determines the vertical transfer efficiency of water within plants and is the key factor in maintaining plant transpiration and water balance. In contrast, radial hydraulic conductivity affects water flow across sections of the root system and affects the water redistribution and reuse (Baca Cabrera et al. 2024). The structure of the xylem network impacts hydraulic conductivity in the axial direction, and the radius of its conduit to the fourth power is proportional to hydraulic conductivity (Liu et al. 2023). Therefore, with sufficient water potential gradient, HR was found to be positively correlated with the diameter of the root conduit size (Hafner et al. 2020). Hence, the inherent tendency of trees to possess thicker root canal diameters elucidates one of the reasons why they exhibit greater predisposition to HR compared to grasses (Fig. 2e). In greenhouse experiments with native Central European tree species, it was observed that oaks with larger conduit diameters and higher hydraulic conductivity redistributed more water when soil water potential remained above the threshold for xylem embolism (Hafner et al. 2017). Similarly, Wan et al. (2000) assessed the HL capacity of three maize hybrids (two drought-tolerant and one drought-susceptible) and found that hybrids with lower root hydraulic conductance (i.e., higher root hydraulic resistance) showed the lowest HL capacity. Root hydraulic conductance is affected by anatomical features of the root vasculature, which have been also suggested to be the cause for a reduced conductivity of the reverse flow compared to the forward flow, as observed in roots of different temperate tree species. This reduced conductivity significantly limited the HR by these trees (Hesse et al. 2019). Additionally, the resistance of roots to radial water transport, including efflux, is influenced by the aquaporin activity (Baca Cabrera et al. 2024). Plants are capable of limiting the reverse flow of water from their roots via the occlusion of aquaporins in their cell membranes (Hafner et al. 2017). A recent study demonstrated that under drought stress, the formation of an impermeable apoplast barrier in plant cell walls restricts the lateral transport of water across the intercellular space, implying that suberin may guide water transport along the root axis (Cantó-Pastor et al. 2024). This process has the potential to promote the redistribution of water within plants by efficiently directing it from deeper soil layers to areas where it is needed, enhancing overall water-use efficiency.

#### Root distribution

Root distribution characteristics, such as root density, rooting depth, and rooting structure are important factors affecting the occurrence of HR (Hultine et al. 2004; Bazihizina et al. 2017; Hafner et al. 2017). Warren et al. (2007) suggested that root density has a greater impact on HR than soil hydraulic conductivity. They found that in two old-growth coniferous forests, the surface area of Douglas-fir fine roots above 100 cm was 25% greater than that of ponderosa pine, and the observed HR peak and magnitude of Douglas-fir were also significantly higher than those of ponderosa pine. This indicated that the spatial variation in HR of Douglas-fir was related to root distribution, with the maximum HR decreasing as the fine root area decreased after saturation (Warren et al. 2007). Hence, it can be concluded that the root density distribution could significantly affect the magnitude of HR (Sallo et al. 2020; Ke et al. 2022). A study conducted in the Pacific Northwest ecosystem revealed that the 0–20 cm soil layer with higher root density exhibited a greater potential to promote HR formation than the 20–60 cm soil layer with a lower root density (Warren et al. 2005). Similarly, Ryel et al. (2002) found that HL is most likely to occur when the root system is more distributed in shallow soil. Furthermore, Wei et al. (2022) found that shallow soils were susceptible to HR in summer, partly due to accelerated water loss through fine root water uptake, which occurred mainly at some distance from the soil surface system in the 30–60 cm layer. Yu & D'Odorico (2014) also discovered that deep soils with greater root density were more favorable to hydraulic descent.

Plants with longer roots are more likely to exhibit HR than those with shorter roots (Rewald et al. 2011; Yang et al. 2022). In contrast to the deep roots of trees, the shallow roots of grasses cannot reach deep moist soil, limiting their potential to lift water from deeper layers to dry surface soil (Fig. 2f) (Espeleta et al. 2004). A comparison between legumes and grasses showed that legumes with deep tap roots can transport water to the deep soil through HD during the rainy season, while in the dry season, they can transport water to the shallow soil through HL (Lee et al. 2018). On the other hand, grasses with shallow roots can only perform HL (Lee et al. 2018). Groundwater supply facilitates root water utilization, and deep roots can perform HR through their ability to reach groundwater, but the water transport capacity of deep roots and shallower groundwater may limit HR (Fig. 2f) (Hultine et al. 2003; Warren et al. 2007). However, Yang et al. (2022) found that the amount of HR in the soil of deep groundwater was relatively small, which could be attributed to the denser distribution of lateral roots in the shallower soil layers, which increased the root-soil contact (Scholz et al. 2008; Neumann and Cardon 2012). Consequently, poor contact between root branches and soil may prevent redistributed water from reaching the rhizosphere (Pregitzer et al. 2002; Ryel et al. 2004; Hafner et al. 2020).

Differences in root structure were also shown to influence the occurrence of HR. Species with dimorphic roots generally exhibited HR more frequently than those with monomorphic roots (Prieto et al. 2012). This provides an explanation for the discrepancy in the HR capacity between trees and grasses, attributed to the divergence in their rooting patterns (Fig. 2d). For instance, in a research on three species in the Chihuahua desert arroyo, plants with deep roots potentially exposed to groundwater did not exhibit reverse sap flow, whereas species with dimorphic roots showed HR (Hultine et al. 2003). Similarly, dimorphic deciduous trees performed HR during both dry and rainy seasons, while monomorphic evergreen trees did not show reverse sap flow in the roots (Scholz et al. 2008). Scholz et al. (2008) observed that due to the lack of lateral roots, monomorphic species might provide some HL in shallow soils through small roots, but the contribution is minimal compared to that of the dimorphic species.

## Plant transpiration

Plant transpiration and stomatal regulation are also important factors affecting the occurrence of HR (Howard et al. 2009; Neumann and Cardon 2012; Bazihizina et al. 2017). High nocturnal transpiration can cause the root system to continue to absorb water during the night after evaporation of a considerable quantity of water, which lowers the water potential of the rhizosphere to a level close to or below the bulk soil water potential, thereby limiting the reverse sap flow (Hultine et al. 2003; Scholz et al. 2008). For instance, internal HR was inhibited in grapes (*Vitis vinifera*) when constant light at night was implemented to keep transpiring (Bauerle et al. 2008). Hultine et al. (2003) demonstrated that reverse sap flow and nocturnal transpiration were negatively correlated in lateral roots of *Fraxinus velutina* and *Juglans major* in shallow dry soils, with maximal reverse flow when transpiration was close to zero. This indicates that nocturnal transpiration may inhibit HR (Domec et al. 2010).

In contrast, when stomata are closed at night and transpiration is very low or inhibited, the water potential within the plant may approach equilibrium with the water potential of the root and soil (Prieto et al. 2012). If there is a water potential gradient between the plant and the soil in relatively dry areas, water would still flow from the roots into these dry soil layers (Prieto et al. 2012). Among the woody species in the Cerrado ecosystem in Brazil, those with the lowest stomatal conductance at night during the dry season have the highest capacity for HL (Scholz et al. 2007). In a greenhouse study conducted by Howard et al. (2009), by bagging *A. tridentata* and *H. anomalus* at night, researchers demonstrated that inhibition of transpiration could significantly increase HR. Similarly, in another experiment on transpiration inhibition of shrubs in different arid lands, rhizosphere water potential in the dry soil layers continued to increase during 48–72 h when shrubs were covered with an opaque plastic fabric (Prieto et al. 2010). The authors indicated that transpiration inhibition and stomatal closure are the triggers for HL (Prieto et al. 2010). During heavy rainfall, when soil moisture is high and plant transpiration requirements are low, plants do not need to absorb water from the wet soil and prevent water loss to the atmosphere (Neumann and Cardon 2012). This reduces the competitive pressure of the dry soil and the atmosphere for water within the plant, making it possible to carry out HR in the deeper but dry soil layers during daytime (Neumann and Cardon 2012). Similarly, under conditions of heavy rain and low transpiration, the upward and downward HR of moist deep and shallow soil layers to the middle layer is promoted (Williams et al. 1993).

However, it is worth noting that nocturnal transpiration does not always negatively impact HR. In fact, under certain conditions, such as when driven by high evaporative demand, nighttime transpiration can also serve as a positive driver of HR (Dawson et al. 2007). A recent study confirmed the phenomenon of evaporation-driven hydraulic redistribution (EDHR) driven by high evaporative demand in *Populus tomentosa* through root sap flow measurements (Liu et al. 2023). Similarly, Dawson et al. (2007) found that among C3 and C4 plants where nocturnal transpiration was common, *sugar maple* exhibited a nocturnal transpiration rate of up to 25% of its daytime rate, which was associated with the greatest HR increase rate. Using structural equation modeling and machine learning algorithms, Yang et al. (2022) analyzed 37 studies and identified a significant positive correlation between HR and daytime transpiration (0–7 mm d^−1^). When transpiration is high during the daytime, it generates a strong suction in stem and root, and soil water depletion is higher, which increases the potential gradient between different layers. The authors concluded that transpiration is the most important driver of HR. This divergent conclusion may stem from the fact that they evaluated the effect of plant transpiration on HR in different species, which differs from previous studies that focused on specific experimental conditions without accounting for the differences between study sites or plant species (Hafner et al. 2020). Thus, the prevalence and variability of transpiration in different environments or among different plant species were not fully assessed.

## Mycorrhizal *fungi*

Mycorrhizal fungi, which form symbiotic associations with plant roots, usually facilitate the movement of water and nutrients through soils by increasing root surface area, thereby enhancing plant access to soil resources (Querejeta et al. 2003; Lehto and Zwiazek 2011; Abdalla and Ahmed 2021; Alagele et al. 2021; Abdalla et al. 2023; Affortit et al. 2024). These fungi can both influence and benefit from HR. For instance, water transported to a certain part of the root system by HR may be further transported through the mycorrhiza, enhancing the spread and utilization of the redistributed water (Brooks et al. 2006). Hafner et al. (2017) suggested that HR in spruce increased under a split-root system due to greater fine root development and enriched mycorrhizal contact, which facilitated higher levels of HR. In another study, under dry soil conditions at night, water transferred from oak to the external mycorrhiza seeps from the mycorrhizal root tips into the soil, increasing the amount of redistributed water available to plants (Querejeta et al. 2003).

The role of mycorrhizal fungi is not limited to passive water transport; they actively participate in enhancing the efficiency of HR. After rainfall, mycorrhizal fungi can enhance the rate of HD by exploring a larger soil contact area to increase water uptake (Prieto et al. 2012). Using deuterium-labeled water, Querejeta et al. (2012) demonstrated that changes in mycelial viability and abundance can modify the pattern of HR in plant roots. This suggests that the mycorrhizal fungi can dynamically adjust to changes in soil moisture and affect the spatial pattern of water redistribution. Additionally, mycorrhizal activity can improve the nutritional status of plants (Querejeta et al. 2003). The water exudation of mycorrhizal fungi in dry soil facilitated nutrient diffusion, reduced the adsorption capacity of soil particles for nutrients, thus increased nutrient uptake by plant roots and indirectly promoted the occurrence of HR. This nutrient mobilization enhances plant growth and resilience, which feeds back positively into the HR process, creating a virtuous cycle of mutual benefit between plant and fungi. Furthermore, mycorrhizal networks facilitate water redistribution between tree species if they share the same mycorrhizal fungal species, thereby contributing to HR on a broader community scale (Egerton-Warburton et al. 2007; Courty Pierre-Emmanuel et al. 2008; Zapater et al. 2011). These findings illustrate the multifaceted role of mycorrhizal fungi in supporting HR. By expanding the reach of hydraulically redistributed water and enhancing both water and nutrient availability, these symbiotic relationships contribute to the overall water balance and nutrient cycling within ecosystems.

## Challenges in detecting hydraulic redistribution

While the occurrence of HR in plants is a common phenomenon, certain experimental conditions can hinder its detection (Thomas et al. 2020). Here are some challenges associated with measuring HR. Firstly, the measurement of HR is constrained by the precision of the detectors. If the redistributed water flux is below the accuracy of the detector probe, HR may not be observed (Meunier et al. 2018). For instance, detecting the impact of HL on soil profile water content is often challenging with conventional soil sensors like TDR. In addition, selecting the appropriate type of soil water sensor and placing it correctly around the root system within the soil profile or in soil layers is crucial to detect the occurrence of HR (Liu et al. 2023). For example, Prieto et al. (2010) detected the HL pattern where soil water potential decreased during the day and increased at night in only one of the four *A. barrelieri* individuals, possibly due to inadequate sensor placement. Similarly, Hultine et al. (2003) failed to detect HR in *Celtis* roots among the three coexisting Arroyo tree species in the Chihuahua desert, potentially due to different root distribution patterns and the smaller roots not being equipped with flow sensors (Cai et al. 2018a).

Another factor influencing HR detection is the plant's capacity for daily water storage. For instance, *Celtis* trees have high daily storage capacities and thus can store water in the stem during the day and release it at night to support transpiration, reducing the need for HR by maintaining a high root water potential compared to soil water potential when the topsoil dried out (Hultine et al. 2003). Although soil moisture sensors can be used for quantitative analysis of redistributed water, the measurement of soil moisture content is often limited to the vicinity surrounding the sensor, which also limits the observation of HR (Thomas et al. 2020). To overcome these limitations, it is necessary to adopt multi-sensor networks, improve sensor performance, and combine soil moisture sensors with artificial intelligence and other technologies to achieve real-time monitoring, transmission, and analysis of data. Furthermore, some studies did not observe HR may because the gradient in water potential between different layers was not sufficient. For instance, Meunier et al. (2018) used stable water isotopes and demonstrated that a strong gradient in soil water potential (ca. −10 MPa in surface and ca. −0.01 MPa in deeper layers in silty soils) would be necessary to observe HR. Similarly, Hayat et al. (2020) employed neutron radiography to ascertain that HR occurred exclusively when the soil water potential of the upper dry soil layers was lower than −0.1 MPa. Similarly, Van Dusschoten et al. (2020) showed that HR began to occur when the water content of sandy soils dried to about 0.05 cm^3^ cm^−3^ (around −0.4 MPa).

Moreover, poor contact between roots and surrounding soils can also result in non-occurrence of HR. Excessively dry soil conditions could lead to the formation of air gaps between the soil and the root system (Duddek et al. 2023) and an increase in root hydrophobicity (Carminati and Vetterlein 2013). The local hydraulic resistance of the soil near the root will increase significantly, reducing the contact area and hydraulic conductivity of the soil-root interface, reducing the diffusion of exuded water from roots, and affecting water transport between the soil and the root system, thus limiting the instances of HR (Couvreur et al. 2020) (see also the discussion below). In *Cerrado* species, the downward sap flow during the transition from dry to wet season was absent, maybe due to the contact of the taproot with the deep moist soil, which reduced the water potential gradient between the upper and lower soil layers (Scholz et al. 2008). Alternatively, after rainfall, high soil hydraulic conductivity could lead to the rapid disappearance of the water potential gradient, preventing HR (Scholz et al. 2008). Zegada-Lizarazu & Iijima (2004) investigated the magnitude of HL in 16 crops using isotope tracing and found that HL was not observed in soybeans, possibly due to dense soil layer impeding water flow and root elongation. However, even at high water potential gradients, Thomas et al. (2020) could not monitor the HR of maize roots using vertical and horizontal root cracking lysimeters, probably due to low soil conductivity and the rapid triggering of compensatory root water uptake, which did not allow enough time for water to diffuse from the root-soil interface into the soil. Moreover, plant survival and genetic variation in varieties also influence HR. Cantó-Pastor et al. (2024) revealed that suberin in plant roots plays a pivotal role in regulating water flow and coping with drought conditions. And they also found that mutants with high root hydraulic conductivity but reduced suberin were more susceptible to water loss under water limitation (Cantó-Pastor et al. 2024). This process may lead to the inability of plants to perform HR. In a field study, Espeleta et al. (2004) noted the loss of HL in *Q. margaretta* and *S. scoparium* due to the death of surface fine roots which resulted from the loss of hydraulic conductivity and root-soil contact. Genetic variation plays a significant role in HL, as demonstrated in a greenhouse experiment by Wan et al. (2000), where HR was observed mainly in drought-tolerant maize compared to drought-susceptible varieties. Aerts et al*.* (1999) have demonstrated that drought resistance exerts an influence on HL ability, with drought-tolerant varieties exhibiting a greater proclivity to develop HR than drought-prone varieties within the same species. This discrepancy may be attributed to the differential expression of regulatory genes, or probably due to differences in root system architecture and plasticity (Wan et al. 2000). This revealed a direct link between HL capacity and drought tolerance, which suggested that HL may exert an important influence on the drought resistance of maize plants.

## Concluding remarks and outlook

In this study, we examined the factors influencing hydraulic redistribution (HR) in various ecosystems, focusing on soil conditions and plant species characteristics. The water potential gradient for HR was around −0.8 to −1.2 MPa, and our analysis indicates that the HR threshold in the drier soil layers was between −1.80 and −0.05 MPa. Compared to sandy and clay soils, loamy soils with their optimal water-holding capacity and permeability significantly promote HR. Root characteristics, including hydraulic architecture and root distribution patterns, also play a crucial role in impacting HR. Dimorphic root species with larger and thicker root canal diameters, which can penetrate deep underground and come into contact with the water table, are more likely to exhibit HR. Mycorrhizal fungi enhance HR by expanding the root uptake area and facilitating water transport (Abdalla et al. 2023; Affortit et al. 2024). Additionally, plant transpiration and stomatal behavior further modulate HR dynamics. Under specific conditions, HR may not be identified due to insufficient soil water potential gradients, inadequate plant physiological regulatory capacity, unfavorable soil texture and/or structures, or limitations in research methods.

Our analysis highlights that while HR can occur in a range of conditions, it is most effective when there is a substantial water potential gradient, proper root-soil contact, and low transpiration rates during dry periods. Given that HR is driven by the water potential gradient, it may be possible to maximize HR by controlling the water potential difference, thereby increasing the amount of water redistributed and improving its efficiency. Further research is therefore required to more accurately determine the water potential threshold under different conditions, including field investigation and modeling predictions.

We also aware that the interactions between these factors are indeed complex and can amplify or diminish the effects of individual components. For example, soil texture can influence root growth patterns, while mycorrhizal associations may modify root architecture and enhance water transport under specific soil conditions. To predict these interactions, we recommend employing a multi-faceted method that integrates experimental data with numerical and statistic modeling techniques to quantify the relative influence of these factors on HR. Consequently, a comprehensive investigation into its internal mechanisms is of paramount importance for developing more drought-resistant crop varieties and enhancing yield. Concerning the influence of root characteristics, further research should examine the distinctions in HR among plants with different root traits, such as root hairs, and root exudation (Cai and Ahmed 2022; Cai et al. 2022). Advanced monitoring techniques will help reveal the physiological effects of HR on plants and ecosystems, aiding the development of appropriate conservation measures to address challenges such as climate change and drought. Studies indicated that, under certain circumstances, tree transpiration through HR can account for approximately 10–70% of total daily transpiration (Hesse et al. 2019), significantly contributing to the water cycle, and affecting water balance and ecological function of the entire ecosystem (Belovitch et al. 2024). Therefore, selecting species with high HR capacity can maximize water use by absorbing water from deep soil layers and redistributing it to surface soils. This process not only provides a stable and sufficient water source for the soil surface transpiration, but also significantly reduces deep soil water loss through evaporation, thereby improving the overall soil water use efficiency. Consequently, HR further enhances the resilience of forests in the face of drought conditions, and provides important ecological services by maintaining the balance and stability of ecosystems. At the same time, promoting HR on a large scale must fully consider its potential environmental impacts and implement appropriate mitigation measures.

Despite considerable efforts to advance our understanding of HR, its specific mechanism remains unclear. Future research should delve deeply into how roots perceive soil water status, optimize species selection, and improve soil structure to adjust HR for better water use efficiency. Additionally, strategies must be developed to adapt HR to global climate changes and drought conditions, as well as the impact of these strategies on plant growth and survival. Given HR’s potential role in maintaining carbon balance, studies should comprehensively examine HR processes at different spatial and temporal scales to quantify their functional role in ecosystems.

## Data Availability

No new data were generated in this study.
